# Availability and components of maternity services according to providers and users perspectives in North Gondar, northwest Ethiopia

**DOI:** 10.1186/1742-4755-10-43

**Published:** 2013-08-23

**Authors:** Abebaw Gebeyehu Worku, Alemayehu Worku Yalew, Mesganaw Fantahun Afework

**Affiliations:** 1 Department of Reproductive Health, Institute of Public Health, University of Gondar, Gondar, Ethiopia; 2 Department of Preventive Medicine, School of Public Health, Addis Ababa University, Addis Ababa, Ethiopia; 3 Department of Reproductive Health and Health Service Management, School of Public Health, Addis Ababa University, Addis Ababa, Ethiopia

**Keywords:** Availability of maternal service, Components of maternity care

## Abstract

**Background:**

The goal of reducing maternal mortality can be achieved when women receive important service components at the time of their maternity care. This study attempted to assess the availability and the components of maternity services according to the perspectives of service users and providers.

**Method:**

A linked facility and population-based survey was conducted over three months (January to March 2012) in North Gondar Zone. Twelve kebeles (clusters) were selected randomly from six districts to identify maternity clients cared for by skilled providers. Then 12 health centers and 3 hospitals utilized by the corresponding cluster population were selected for facility survey. Interview with facility managers/heads, providers and clients and observations were used for data collection. Data were entered using Epi Info and were exported to SPSS software for analysis.

**Results:**

Antenatal and delivery care were available in most of the visited facilities. However, the majority of them were not fully functioning for EmOC according to their level. Signal functions including administration of anticonvulsants and assisted vaginal delivery were missing in seven and five of the 12 health centers, respectively. Only one hospital met the criteria for comprehensive emergency obstetric care (performed cesarean section). Only 24% of the providers used partograph consistently. About 538 (32.3%) and 231 (13.8%) of the women received antenatal and delivery care from skilled providers, respectively. Most of the services were at health centers by nurses/midwives. At the time of the antenatal care, women received the important components of the services (percentage of users in bracket) like blood pressure checkup (79%), urine testing (35%), tetanus immunization (45%), iron supplementation (64%), birth preparedness counseling (51%) and HIV testing (71%). During delivery, 80% had their blood pressure measured, 78% were informed on labor progress, 89% had auscultation of fetal heartbeat, 80% took drugs to prevent bleeding and 78% had counseling on early & exclusive breast-feeding.

**Conclusion:**

Antenatal and delivery care were available in most of the visited facilities. However, important components of both the routine and emergency maternity care services were incomplete. Improving the functional capacity of health facilities for the delivery of routine maternity and EmOC services are needed.

## Background

Utilization of maternal health services by skilled providers, a proximate indicator of maternal survival, is very low in Ethiopia. According to the recent (2011) Ethiopian Demographic and Health Survey (EDHS) report, only 34%, 10% and 6% women have antenatal (ANC), delivery, and postnatal care by a skilled provider, respectively [1].

One of the key factors for the utilization of skilled maternal care is access to and availability of health care facilities. Evidences from many developing countries, including Mali, South Africa, Zambia, Paraguay, Uganda and Tanzania indicate that the proximity of maternal health services to users and a reliable transportation system to link the community and health facilities are critical for maternal service utilization [2-5]. The availability of basic and comprehensive emergency obstetric care (signal functions) in a health care system are crucial for maternal survival [6]. Signal functions, including parenteral antibiotics, anticonvulsants and oxytocics, manual removal of placenta, removal of retained products, assisted vaginal delivery and neonatal resuscitation (at basic emergency obstetric care facility) with the addition of cesarean delivery and blood transfusion (at comprehensive emergency obstetric care facility) used to treat direct obstetric complications cause the majority of maternal deaths [7].

In order to have a better health care access, Ethiopia has introduced a three-tier health care delivery system. The first level is the primary health care unit or district health system comprising a primary (district) hospital (to cover 60,000-100,000 people), health centers (1/15,000-25,000 population), and their satellite health posts connected to each other by a referral system. Level two is a general hospital covering a population of 1–1.5 million people, and level three is a specialized hospital covering a population of 3.5-5 million people [8]. District level facilities are organized to provide obstetric first aid at health post levels with early referral to health centers that can provide basic emergency/essential obstetric care and further referral to district hospitals for comprehensive emergency/essential obstetric care.

Once the mother arrives at the health facility, she has to have access to a medical professional who has the skills and equipment to give the necessary services. However, in many developing countries, health facilities do not perform their expected functions according to their level. For instance, a study on health facilities in 13 countries found out that most hospitals did not provide life saving services fully. In the study, assisted vaginal delivery was missing in 42%, 73%, 38%, and 19% of the hospitals in Benin, Mali, Morocco, and Uganda, respectively. The removal of retained products was missing in 12%, 9% and 12% of the hospitals in Benin, Mali and Morocco, respectively [9]. Similar studies revealed the fact that facilities were not performing what they were supposed to [10].

Studies showed that many facilities suffered from staff training deficiencies that hindered the provision of specific services. In many settings, midlevel providers such as midwives were not trained or authorized to perform some of the key procedures expected from a skilled attendant. A study in Benin, Ecuador, Jamaica, and Rwanda showed a wide gap between current evidence based standards and current levels of provider competence. In the study, providers answered 55.8% of the knowledge questions and performed 48.2% of the skills steps correctly [11,12]. Pre-service training is also not a guarantee. It is recommended that maternity care providers receive refresher training or updates in midwifery every three to five years [13].

In Ethiopia, many of the previous studies have reported ANC, delivery and postnatal care coverage as utilization of maternal services. However, achieving the goals of maternal health care (reduction in morbidity and mortality) depends more on utilization of important service components (better quality of care) than high coverage of the services. Essential interventions (service components) provided before pregnancy, during pregnancy, delivery, and postpartum are outlined in the WHO documents and lancet article [14,15]. In the country, there is limited evidence about specific service components and the performance of health facilities at the time of maternity care. Therefore, the question, “What kind of service is available and is delivered by a skilled provider at the time of maternity care?” needs an appropriate answer for planning as well as, monitoring maternal health services. This study was planned to assess the availability and components of maternity services in both routine and emergency situations according to providers and users perspectives in North Gondar.

## Methods

Two surveys, including the facility survey, to collect information from the providers and clients, and the interviews with the population to gather information from users’ were carried out concurrently. As suggested in the manuals [16], the facility survey was linked to the population-based survey sample areas. The study was conducted over three months (January - March 2012) in North Gondar Zone. The zone has 22 ‘woredas’ (districts). According to the figures from the central statistical agency, 2007, this zone had an estimated total population of 2,921,470, about 84.1% of which were rural dwellers [17]. In the zone, there is only one referral hospital with a comprehensive emergency obstetric care. There are also about a hundred health centers and two district hospitals. The zone, which is the largest of the region, has a difficult topography.

In order to get a sufficient number of maternal care users, a total of twelve clusters or kebeles (the lowest administrative units having clear geographical boundary and administrations) were selected randomly from six districts, namely Dembia, Lay Armachiho, West Belessa, Metema, Debark and Dabat. All women who had births one-year preceding the survey and who received maternal care during pregnancy and delivery were included in the sample. Based on 34% and 10% coverage of skilled antenatal and delivery care [1], the sample size was determined on the assumption of 95% confidence level and 4% of worst acceptable error. In the selected clusters, 538 and 231 of the eligible women were skilled antenatal and delivery care users.

One of the options for sampling facilities in a linked survey is determine the nearest facility, or facilities, to each population-based survey cluster and conduct the facility survey in all identified. In this approach, the sample size is improved by assuring that the number of facilities is no less than the number of population-based clusters. Based on this recommendation, we have identified one basic essential obstetric care facility (health center utilized by the cluster population) for each selected cluster (kebele). Twelve health centers and three hospitals serving the selected kebele population were included for the facility survey. Interview with managers/heads of health institutions and observation were used to collect data related to the availability, readiness, and functioning of facilities for the provision of skilled maternal care. During observation, a checklist containing infrastructure, equipment, drugs and supplies, and laboratory tests was prepared. Based on adequacy and functional status, most of the findings were rated as 0) not available (not functional), 1) available but unsatisfactory and 2) available and functional. In addition, all midlevel skilled care providers who were directly involved in maternity care at these health centers and district hospitals (38 skilled providers) were interviewed about their training and skills for different types of procedures. A skilled attendant is an accredited health professional who has been educated and trained to proficiency in the skills needed to manage normal (uncomplicated) pregnancies, childbirth and the immediate postnatal period, and in the identification, management and referral of complications in women and newborns [18,19]. In this study, skilled providers included midwives, nurses, health officers and doctors. Health officers are those trained for four years to get their Bachelors degree and they work as clinician in the rural set up (health centers).

Thirty-six data collectors and supervisors (2 data collectors and 1 supervisor for each kebele) were trained and deployed for identifying and interviewing maternal care users. The interviews were conducted using the local language (Amharic) after the questionnaire was pretested for cultural appropriateness and clarity. To conduct the facility-based survey, 14 experienced health professionals were recruited. After the appropriate coding, the data was entered in Epi Info version 3.5.3 software, and exported to SPSS software for analysis.

Before the commencement of the study, ethical clearance was obtained from the Institutional Review Board of the College of Health Sciences, Addis Ababa University. During data collection, the study participants were asked for consent and informed to interrupt the interview on desire. All participants signed written agreements (consent forms). To ensure confidentiality, names were not used in depicting the results of the study.

## Results

### Availability of the routine maternal services at health centers and hospitals

All facilities (hospitals and health centers) reported that they had ANC and family planning (FP) services. Most of the facilities, except one health center, claimed that they had delivery and PMTCT services, but one-fourth of the visited health centers did not have postnatal care in the first 7 days. Five out of the 12 health centers and all hospitals had safe abortion services (Table 1).

**Table 1:** Availability of maternal services and laboratory tests in basic and comprehensive obstetric care facilities (selected health centers and all hospitals) in North Gondar, 2012

**Type of services available in the facility**	**Number of visited facilities**		
	**Health centers (12)**	**Hospitals (3)**	**Total (15)**
***Availability of selected maternity related services***			
Antenatal care	12	3	15
Delivery care	11	3	14
Postnatal care in the first 7 days	9	3	12
Family planning	12	3	15
VCT	10	3	13
PMTCT	11	3	14
Safe abortion	5	3	8
Maternity service for 24 hrs and 7 days a week	9	3	12
Working ambulance (referral service)	3	2	5
***Signal functions to treat major obstetric complications***			
Parenteral antibiotics	11	3	14
Parenteral oxytocics	11	3	14
Anticonvulsants	5	3	8
Manual removal of placenta	9	3	12
Removal of retained products	10	3	13
Assisted vaginal delivery	7	2	9
Cesarean section	0	1	1
Blood transfusion	0	2	2
***Availability of selected laboratory tests***			
Syphilis testing (VDRL)	0	3	3
Hemoglobin test	5	3	8
Urine test-protienuria	6	3	9
Blood film-malaria	11	3	14
HIV test	12	3	15
Cross match tests	5	2	7

However, findings from the interview with skilled maternal care users indicated that out of the 1668 women who gave birth one year preceding the survey, only 538 (32.3%) and 231 (13.8%) received antenatal and delivery care from skilled providers, respectively. Of the total users of the ANC service, 86% received their care at health centers, and most (92%) of the users were served by nurses or midwives. Similarly, three-fourths of the births took place at health centers assisted by nurses or midwives.

None of the health centers visited had laboratory services for testing syphilis. Moreover, many health centers did not have laboratory tests for hemoglobin, urine for protienuria, and cross match (Table 1).

### Emergency obstetric care signal functions

A total of five health centers and all three hospitals fulfilled the criteria for signal functions for basic emergency obstetric care, but only Gondar Hospital fulfilled all the signal functions for comprehensive emergency obstetric care. Of the signal functions, parenteral antibiotics and oxytocics were practiced by all the facilities visited except one health center. However, important functions, including the administration of anticonvulsants and assisted vaginal delivery were missing in three-fifths and two-fifths of the health centers, respectively. Only one-fourth of the health centers had working ambulances to give referral service (Table 1).

### Important service components received during antenatal and delivery care

At the time of antenatal care, some women received important components of services (percentage of users in bracket) like blood pressure checkup (79%), urine testing (35%) tetanus immunization (45%), iron supplementation (64%), birth preparedness counseling (51%), saving money for possible complication (45.2%), and HIV testing (71%).

Among women who have delivery by a skilled attendant, about 80% had their blood pressure measured, 78% informed on labor progress, 89% had auscultation of fetal heartbeat, 80% took drugs to prevent bleeding and 78% had counseling on early & exclusive breast-feeding. Weight was measured and vaccine was provided for babies of 67% and 69% of mothers, respectively. There are some differences in providing services by type of providers (Table 2).

**Table 2:** Important service components received by women during their ANC visit and delivery care by types of skilled attendants, North Gondar, 2012

**Types of services**	*Service users by qualification of their provider							
	**Doctor**		**Health officer**		**Midwife/Nurse**		**Total**	
	**Yes**	**%**	**Yes**	**%**	**Yes**	**%**	**Yes**	**%**
**Services received by women during their ANC visits by a skilled attendant**								
Medical history evaluated	19	82.6	19	86.4	425	86.2	463	86.1
Blood pressure measured	19	82.6	18	81.8	390	79.1	427	79.4
Weight measured	18	78.3	20	90.9	375	76.1	413	76.8
Blood sample taken	17	73.9	19	86.4	349	70.8	385	71.6
Urine sample taken	9	39.1	11	50.0	168	34.1	188	34.9
TT immunization provided	9	39.1	10	45.5	223	44.8	242	45.0
Anti malaria drugs provided	9	39.1	10	45.5	211	42.8	230	42.8
Iron/folic acid provided	15	65.2	15	68.2	315	63.9	345	64.1
Expected due date told	10	43.5	12	54.5	244	49.5	266	49.4
Nutrition advise provided	16	69.6	16	72.7	302	61.3	334	62.1
Fetal growth updated	10	43.5	12	54.5	292	59.2	314	58.4
Birth preparedness discussed	11	47.8	11	50.0	250	50.7	272	50.6
Needed materials for delivery discussed	9	39.1	12	54.5	242	49.1	263	48.9
Place of birth discussed	15	65.2	15	68.2	300	60.9	330	61.3
Advised to go health facility at time of birth	16	69.6	14	63.6	304	61.7	334	62.1
Saving funds discussed	7	30.4	12	54.5	224	45.4	243	45.2
Discussion on danger signs	9	39.1	14	63.6	258	52.3	281	52.2
Discussions on complications during delivery or postpartum	11	47.8	12	54.5	254	51.5	277	51.5
Discussed to go Health facility at time of complication	15	65.2	14	63.6	254	51.5	283	52.6
Discussed to prepare blood donor	9	39.1	10	45.5	183	37.1	202	37.5
Discussed on HIV testing	17	73.9	19	86.4	348	70.6	384	71.4
Discussed about PMTCT	13	56.5	17	77.3	347	70.4	377	70.1
Discussed about other STIs	8	34.8	8	36.4	233	47.3	249	46.3
Total	23	100	22	100	493	100	538	100
**Services received by women during their delivery care by a skilled attendant**								
General health assessment	28	80.0	20	87.0	151	87.3	199	86.1
Blood pressure & other vital signs	29	82.9	17	73.9	138	79.8	184	79.7
Per vaginal examination	28	80.0	17	73.9	140	80.9	185	80.1
Abdominal examination	31	88.6	21	91.3	158	91.3	210	90.9
Auscultation of fetal heart beat	31	88.6	21	91.3	153	88.4	205	88.7
Control bleeding by drugs	28	80.0	22	95.7	134	77.5	184	79.7
Information on progress of labor	28	80.0	17	73.9	136	78.6	181	78.4
Keep comfort of mother	29	82.9	20	87.0	132	76.3	181	78.4
Give care for the baby	26	74.3	17	73.9	124	71.7	167	72.3
Measure and record baby weight	18	51.4	15	65.2	121	69.9	154	66.7
Give vaccine for baby	27	77.1	14	60.9	118	68.2	159	68.8
Advise about breast feeding	27	77.1	17	73.9	137	79.2	181	78.4
Advise on infant care	21	60.0	12	52.2	94	54.9	127	55.0
Total	35	100	23	100	173	100	231	100

*Number of service users who said “Yes” during the interview and percentage within type of their provider was calculated.

### Availability of essential drugs, supplies and equipment

The observation of facilities showed that essential drugs, including antibiotics, intravenous fluids, vaccines and contraceptives were available in the 15 selected facilities. Supplies, like gloves and syringes were also adequate. However, pieces of equipment (number of facilities out of 15 facilities in brackets), including thermometers (5), sphygmomanometers (7), foetoscopes (2), delivery set (5), episiotomy set (5), vacuum extractor (10), blank partograph (7), MVA set (8), bag and mask for neonatal resuscitation (7), sterilizer (5) and refrigerator (8) were either missing or nonfunctional at the time of visiting facilities. Similarly, private consultation rooms (5), delivery room (5), toilet facilities (7), water supply (8) and drainage system in the labor room (13) were unsatisfactory.

### Providers training and skills to provide maternal services

Interview with providers directly involved in (assigned to) maternity care at basic obstetric care facilities (health centers and district hospitals) was done to assess providers training and their skills. From the interview, it was found that adequate training or on-the-job experience was obtained by the majority of providers for different types of procedures, including ANC risk screening (89%), partograph utilization (79%), intra-venous infusion (97%), episiotomy (87%), repairing vaginal tears (82%), active management of third stage (97%) and manual removal of placenta (92%). However, only a limited proportion of providers had adequate training or experience on important procedures, like assisted vaginal delivery (vacuum extraction) (45%), manual vacuum aspiration (45%) and evacuation and curettage (26%) (Table 3).

**Table 3:** Number and percent of skilled providers having training or on-the-job experience for different types of procedures, North Gondar, 2012

**Type of procedures**	**Qualification of provider**					
	**Health officer (n = 9)**		**Nurse/midwife (n = 29)**		**Total (n = 38)**	
	**n**	**%**	**n**	**%**	**n**	**%**
ANC risk screening	8	88.9	26	89.7	34	89.5
Partograph utilization	7	77.8	23	79.3	30	78.9
IV infusion	9	100	28	96.6	37	97.4
Speculum examination	7	77.8	15	51.7	22	57.9
Bimanual examination	8	88.9	19	65.5	27	71.1
Compress uterus	7	77.8	20	69.0	27	71.1
External version	1	11.1	4	13.8	5	13.2
Internal version	0	0	3	10.3	3	7.9
Episiotomy	9	100	24	82.8	33	86.8
Repairing cervical laceration	0	0	5	17.2	5	13.2
Repairing vaginal tears	8	88.9	23	79.3	31	81.6
Repairing third/fourth degree tear	1	11.1	11	37.9	12	31.6
Active management of third stage	9	100	28	96.6	37	97.4
Manual removal of placenta	9	100	26	89.7	35	92.1
Assisted vaginal delivery (Vacuum extraction)	6	66.7	11	37.9	17	44.7
Manual vacuum aspiration	6	66.7	11	37.9	17	44.7
Evacuation and curettage	5	55.6	5	17.2	10	26.3

Most of the interviewed providers claimed that they routinely performed ANC screening procedures, like checking blood pressure, anemia, fetal heartbeat, malaria and HIV. However, only two-fifths, most of them in the hospitals, performed VDRL tests. At the time of delivery care, about 42% of the providers said that they were checking laboring mothers frequently according to the recommended standard and only 24% of them were using partograph consistently. Many providers (44%) never introduced themselves to their clients. Nonetheless, they routinely performed other procedures, like protecting privacy, explaining procedures, informing about the progress of labor and possible outcomes, as well as comforting the laboring mother. The providers did most of the important procedures and counseling at the time of postpartum care but only 68% of them did vaginal examination. Moreover, many providers reported that they had the skills to manage common obstetric complications, like preeclampsia (68%), severe bleeding (95%), retained placenta (97%), and post-abortion complications (68%) (Table 4).

**Table 4:** Reports of skilled care providers in the past three months performing the following procedures to women who present for maternity care, North Gondar, 2012

**Types of services**	**Qualification of provider**					
	**Health officer (n = 9)**		**Nurse/midwife (n = 29)**		**Total (n = 38)**	
	**n**	**%**	**n**	**%**	**n**	**%**
**Routinely perform the following steps/check-up to women who present for antenatal care**						
Take medical history	9	100	29	100	38	100
Check BP	9	100	29	100	38	100
Check anemia	9	100	29	100	38	100
Check fetal heart beat	9	100	29	100	38	100
Provide iron/folic acid supplement	8	88.9	27	93.1	35	92.1
Check malaria	9	100	28	96.6	37	97.4
Test proteinuria	7	77.8	20	69.0	27	71.1
Test syphilis	4	44.4	11	37.9	15	39.5
Counsel on self care	9	100	27	93.1	36	94.7
Counsel on delivery plan	9	100	29	100	38	100
Discuss nutrition	9	100	29	100	38	100
Tell due date	9	100	29	100	38	100
Discuss funds for expense	6	66.7	26	89.7	32	84.2
Discuss on transport	9	100	23	79.3	32	84.2
Discuss on blood donors	4	44.4	18	62.1	22	57.9
Discuss VCT	9	100	27	93.1	36	94.7
Discuss PMTCT	9	100	26	89.7	35	92.1
**Routinely perform the following at the time of delivery care**						
Checking laboring mother frequently according to the recommended standard	5	55.6	11	37.9	16	42.1
Partograph use at least once	5	55.6	14	48.3	19	50.0
Partograph use almost always	4	44.4	5	17.2	9	23.7
Introducing for client	4	44.4	18	62.1	22	57.9
Allow family to enter	9	100	25	86.2	34	89.5
Protecting privacy by screen/clothes	9	100	28	96.6	37	97.4
Explain procedures	8	88.9	27	93.1	35	92.1
Tell the progress of labor	9	100	28	96.6	37	97.4
Tell about possible outcomes	8	88.9	26	89.7	34	89.5
Keep comfort of laboring mother	9	100	29	100	38	100
Advise to have postpartum care (1^st^ wk)	6	66.7	15	51.7	21	55.3
**Routinely perform the following at the time of postpartum care**						
Abdominal examination	9	100	24	82.8	33	86.8
Vaginal examination	8	88.9	18	62.1	26	68.4
Checking BP	9	100	29	100	38	100
Assessing abnormal bleeding	9	100	29	100	38	100
Inspection of lochia	9	100	26	89.7	35	92.1
Discussing FP	9	100	29	100	38	100
Advise BF	9	100	29	100	38	100
Checking baby	9	100	27	93.1	36	94.7
**Management of selected complications**						
Preclampsia	8	88.9	18	62.1	26	68.4
Severe bleeding	9	100	27	93.1	36	94.7
Retained placenta	9	100	28	96.6	37	97.4
Post abortion care	7	77.8	19	65.5	26	68.4

## Discussion

The study showed that maternity and related services, including antenatal and delivery care were available in most of the visited facilities. However, the majority of the facilities were not fully functioning for emergency obstetric care (EmOC) according to their level. In health centers (basic EmOC facilities), parenteral anticonvulsants and assisted vaginal delivery were often missing. In the hospitals, cesarean section and blood transfusion were not available. Evidences showed that these functions were commonly unavailable in many other areas [9,20]. Only one hospital met the criteria for comprehensive EmOC (performed cesarean section). This hospital is serving about three million people of the zone and the surrounding areas. It has been recommended that for every 500,000 population at least one comprehensive EmOC facility is required [7]. According to this standard, more (about six) facilities fulfilling the signal functions of comprehensive EmOC are needed. The finding implies that many mothers living in distant (>100 km) districts were obliged to travel long distance to get a functional facility at the time of complications. The findings also indicated that many pregnant women who developed obstetric complications were not benefiting from their care.

Understanding how facilities are actually functioning is more useful than how they are supposed to function. Functioning obstetric facility means performing the essential services for normal situations and performing each signal functions for complications, and these services are available 24 hours a day and 7 days a week. Such functions require enabling environment like facilities and skilled professionals. In many of the visited facilities, important pieces of equipment were either missing or not functional. Most of the health centers also lacked important laboratory tests such as VDRL, hemoglobin, urine protein, and cross match. The absence of such tests has a clear effect on screening services during maternity care. This was a common challenge identified by other studies [21]. Moreover, many of the interviewed midlevel providers had no adequate training on important procedures, like assisted vaginal delivery (vacuum extraction), manual vacuum aspiration or evacuation and curettage. The use of partograph to monitor labor progress helps to take life saving action in a timely manner. However, few providers used partograph consistently. In addition, about one-third had no skill to manage preeclampsia and post-abortion complications. Training deficiencies and skill retention problems were among the major bottlenecks to have quality maternal care in many developing countries [11,22].

Providing incomplete (poor quality) maternal service had damaging effect on health-seeking behavior of mothers. The linked data analysis, as continuation of this study, indicated that utilization of skilled maternal care by individual woman depends on the joint effect of individual, household, communal and facility characteristics. Our analysis indicated that the presence of all the six signal functions in the nearby basic essential obstetric care facility (health center) positively contributed to the utilization of all indicators of skilled maternal services, and its effect was significant on skilled attendance rate [23].

According to the interview with mothers who received antenatal care from skilled providers, most of their service was at health centers by nurses or midwives. They rarely used doctors or specialists. After criticisms on the traditional ANC (the risk approach), the new approach that is focused antenatal care emphasizes the quality of service. In this study, the implementations of the important components of ANC services were incomplete. Many mothers did not receive various antenatal screening services. Tetanus immunization, iron supplementation, counseling and advice on nutrition, birth preparedness and readiness for possible complication were lower than expected. The identified gap in all areas of service components indicated that the current antenatal care service could be challenged to achieve the goal of focused ANC.

Like the ANC, many women who had delivery care by a skilled attendant did not receive important services. To more than 20% of the mothers’ services, like blood pressure measurement, information on labor progress, and drugs for preventing bleeding were not provided. For more than 30% of the mothers, baby weight was not measured and vaccine was not provided. Providers also paid little attention to counseling and advice on different types of maternal and childcare. In this regard, the poor skills of providers may be the main causes of the unsatisfactory services. In many developing countries, competency and skill retention of providers are the major concerns for skilled birth attendance [12,24].

This study has had certain limitations. During the interview, for example, women faced difficulties to differentiate types of skilled providers. To minimize the problem, data collectors did further clarifications by collecting information on types of providers from health institutions that gave the services. Because of the cross-sectional design of the study, recall and interviewer bias were also potential limitations. However, numerous scientific procedures were employed to minimize the possible effects. To reduce the recall bias, for instance, only women who gave birth in the last one year were included.

In conclusion, maternity services including antenatal and delivery care by a skilled provider were available in most of the visited facilities. However, components of both the routine and emergency maternity care services were incomplete. The majority of the facilities were not fully functioning for EmOC according to their level. The implementations of the important components of ANC and delivery services were incomplete and thus, many mothers did not receive various components of the services. The findings suggested that providing incomplete (poor quality) maternal service is the country’s major challenge to achieve the MDG target of reducing maternal mortality. Improving the functional capacity of health facilities for the delivery of routine maternity and EmOC services are needed. According to evidence of other countries, it will be good to focus on interventions, like renovations and upgrading facilities, provision of essential equipment, and on-job training of staff [18,25].

## Competing interests

The authors declare that they have no competing interests.

## Authors’ contributions

All authors contributed equally during the process of proposal development. AG and AW participated in data collection and analysis. AG prepared the draft. Then AW and MF revised drafts of the paper. All authors read and approved the final manuscript.
